# 

**Published:** 1820-06-01

**Authors:** 


### Contents.

Art. I.

APOPLEXY.

Dr. Cooke on Nervous Diseases — Dr. Abercrombie on Apoplexy — Dr. Bricheteau on Apoplexy — Dr. Rochoux on Apoplexy — Dr. Serres on Apoplexy 1

II.

Mr. Evans's Pathological and Practical Remarks on Ulcerations of the Genital Organs - - - - - - - - - - 28

### CONTENTS.

III.

Dr. HAMILTON's Observations on the Use and Abuse of Mercurial Medicines in various Diseases - - - - 41

IV.

Annuaire Medico-Chirurgicale des Hopitaux et Hospices Civils de Paris:—Art. 1. M. Dupuytren on Dislocations of the Ancle-Joint (with an Engraving) - - - - 59

V.

1. Mr. GUTHRIE on the Operations for the Formation of an Artificial Pupil, &c.—2. Sir WILLIAM ADAMS's Treatise on Artificial Pupil, &c. - - - - - - - 71

VI.

Dr. PARIS's Pharmacologia, or History of Medicinal Substances, with the View to establish the Art of prescribing extemporaneous Formulæ, &c. &c. - - - 80

VII.

SUPPLEMENTAL REVIEW of PRACTICAL MEDICINE:—

1. Mr. Keat's Case of Bony Tumour of the Skull - - 98

2. Dr. Kluyskins on Ophthalmia - - - - - - - - 100

3. M. Carré's Case of Gastrotomy - - - - - - - 101

4. Mr. Windsor on Extirpation of the Uterus - - - 104

5. Effects of suppressed Menstruation - - - - - - - 107

6. Mr. Latouche on Rupture of the Uterus - - - - 108

7. Dr. Marcet on Nephritis Calculosa - - - - - - - 109

8. Mr. Benjamin Hutchinson on Tic Douloureux - - 111

9. Dr. Blundell on the Transfusion of Blood - - - - 117

10. Mr. Farr on Scrofula - - - - - - - - - - - - 119

11. Dr. Colles on the Lymphatic Glands - - - - - - 122

12. Dr. Davy on Post Mortem Changes - - - - - - 122

13. Drs. Millingen and Luscombe on Military Hygiene - 125

14. Dr. Porter's Liquor Morphii Citratis - - - - - - 127

VIII.

Dr. W. P. DEWES' Essay on Puerperal Convulsions - - - 128

IX.

EPIDEMIC FEVER.

1. Dr. GRATTAN's Reports on the Epidemic Fevers of Ireland 139
2. Dr. CRAMPTON's Medical Report of the Fever Department in Steevens' Hospital, &c. - - - - - - - - - - 145

X.

Dr. WEATHERHEAD's Treatise on Infantile and Adult Rickets 153

INTELLIGENCE - - - - - - - - - - - - - - - 156—160

---

☞ This leaf is to be carefully preserved for binding up, as an integral part of the Table of Contents, with the Volume.

